# An effector of *Erysiphe necator* translocates to chloroplasts and plasma membrane to suppress host immunity in grapevine

**DOI:** 10.1093/hr/uhad163

**Published:** 2023-08-16

**Authors:** Bo Mu, Zhaolin Teng, Ruixin Tang, Mengjiao Lu, Jinfu Chen, Xiangnan Xu, Ying-Qiang Wen

**Affiliations:** State Key Laboratory of Crop Stress Biology for Arid Areas, College of Horticulture, Northwest A&F University, Yangling 712100, Shaanxi, China; Key Laboratory of Horticultural Plant Biology and Germplasm Innovation in Northwest China, Ministry of Agriculture and Rural Affairs, Yangling 712100, Shaanxi, China; State Key Laboratory of Crop Stress Biology for Arid Areas, College of Horticulture, Northwest A&F University, Yangling 712100, Shaanxi, China; Key Laboratory of Horticultural Plant Biology and Germplasm Innovation in Northwest China, Ministry of Agriculture and Rural Affairs, Yangling 712100, Shaanxi, China; State Key Laboratory of Crop Stress Biology for Arid Areas, College of Horticulture, Northwest A&F University, Yangling 712100, Shaanxi, China; Key Laboratory of Horticultural Plant Biology and Germplasm Innovation in Northwest China, Ministry of Agriculture and Rural Affairs, Yangling 712100, Shaanxi, China; State Key Laboratory of Crop Stress Biology for Arid Areas, College of Horticulture, Northwest A&F University, Yangling 712100, Shaanxi, China; Key Laboratory of Horticultural Plant Biology and Germplasm Innovation in Northwest China, Ministry of Agriculture and Rural Affairs, Yangling 712100, Shaanxi, China; State Key Laboratory of Crop Stress Biology for Arid Areas, College of Horticulture, Northwest A&F University, Yangling 712100, Shaanxi, China; Key Laboratory of Horticultural Plant Biology and Germplasm Innovation in Northwest China, Ministry of Agriculture and Rural Affairs, Yangling 712100, Shaanxi, China; College of Water Resources and Architectural Engineering, Northwest A&F University, Weihui Road 23, Yangling 712100, Shaanxi, China; State Key Laboratory of Crop Stress Biology for Arid Areas, College of Horticulture, Northwest A&F University, Yangling 712100, Shaanxi, China; Key Laboratory of Horticultural Plant Biology and Germplasm Innovation in Northwest China, Ministry of Agriculture and Rural Affairs, Yangling 712100, Shaanxi, China

## Abstract

The powdery mildew (*Erysiphe necator*) is a prevalent pathogen hampering grapevine growth in the vineyard. An arsenal of candidate secreted effector proteins (CSEPs) was encoded in the *E. necator* genome, but it is largely unclear what role CSEPs plays during the *E. necator* infection. In the present study, we identified a secreted effector CSEP080 of *E. necator*, which was located in plant chloroplasts and plasma membrane. Transient expressing *CSEP080* promotes plant photosynthesis and inhibits INF1-induced cell death in tobacco leaves. We found that *CSEP080* was a necessary effector for the *E. necator* pathogenicity, which interacted with grapevine chloroplast protein VviB6f (cytochrome b6-f complex iron–sulfur subunit), affecting plant photosynthesis. Transient silencing VviB6f increased the plant hydrogen peroxide production, and the plant resistance to powdery mildew. In addition, CSEP080 manipulated the VviPE (pectinesterase) to promote pectin degradation. Our results demonstrated the molecular mechanisms that an effector of *E. necator* translocates to host chloroplasts and plasma membrane, which suppresses with the grapevine immunity system by targeting the chloroplast protein VviB6f to suppress hydrogen peroxide accumulation and manipulating VviPE to promote pectin degradation.

## Introduction

The biotic stresses threatening plant growth are numerous, and one of the most common pathogens is fungi. Plants have developed a multi-layer immune system to defend against pathogens [1, 2]. There are two types of receptors, pattern-recognition receptors (PRRs), locating in the plasma membrane and nucleotide-binding leucine-rich repeat receptors (NLRs), locating in the intracellular [3, 4]. When pathogens are inoculated on the plant, pathogen-associated molecular patterns (PAMPs) would be recognized by plant PRRs, which triggers the plant immunity (PTI) [5]. Then, the plant would activate the calcium influx, the mitogen-activated protein kinases (MAPKs) cascades signaling, and reactive oxygen species (ROS) production, as well as induce the expression of defense-related genes and callose deposition, inhibiting the pathogens infection [6, 7]. To compete with the PTI, pathogens would secrete an arsenal of effectors into plant cells through the secretory system or haustorium to interact with PRRs, and block the transmission of defense signals [6, 8]. However, pathogen effectors can be recognized by plant NLRs in some resistant species, which triggers plant immunity (ETI) [9, 10]. ETI is a stronger and longer lasting plant hypersensitivity response (HR) compared to PTI, working through triggering programmed cell death (PCD) [7]. Under these circumstances, pathogens’ effectors will evolve to escape the NLRs’ monitor for avoiding the ETI [11, 12]. Meanwhile, the NLRs also evolve to strengthen the perception of effectors [1, 4, 13]. Hence, plants and pathogens are in an arms race.

Plant chloroplast is the light-harvesting organelle for photosynthesis and sugar synthesis. It is also a place where plants activate defense signals by ROS production and primary metabolisms such as nitrous oxide (NO) and salicylic acid (SA) [14–17]. In the interaction between plants and pathogens, plant chloroplasts play an indispensable role in regulating plant immunity in numerous species [18–20]. For instance, rice stripe virus encodes the *NSvc4* effector to suppress chloroplast-mediated immunity by targeting the host chloroplast protein [21]. The Irish potato uses chloroplasts’ interconnected haustoria to form dynamic organelle clusters against the famine pathogen *Phytophthora infestans* infection [22]. For grapevine, a candidate effector in *Plasmopara viticola* named RXLR31154 could interact with *Vitis piasezkii* chloroplast protein VpPsbP, reducing the H_2_O_2_ accumulation and the ^1^O_2_ signaling pathway, which promotes *P. viticola* infection [23]. Furthermore, other scholars identified a haustorium-specific protein (Pst_12806) from *Puccinia striiformisf. sp. tritici* (*Pst*), which could interact with the C-terminal Rieske domain of the wheat TaISP protein when translocated into chloroplasts, reducing the callose deposition, the expression of defense-related genes and the ROS production, benefitting *Pst* infection [24]. In addition, there were two low sequence similarities effectors both interacting with TaISP to limit host ROS accumulation and promote fungal infection [25]. However, it was not known what part the plant chloroplasts take in plant resistance to powdery mildew.

Pectin is a vital part of cell composition and plant defenses [26]. Plants would be more susceptible to various pathogens if pectin biosynthesis was impaired. Pectin affects pathogen penetration due to impaired cell-wall integrity (CWI) [27]. Pectin lyase regulates cotton resistance to Verticillium wilt through inducing cell apoptosis of *Verticillium dahliae* [28]. In addition, pectin-derived oligogalacturonide can downregulate the expression of *lncRNA2* and *GbPG12* to accelerate pectin accumulation, which enhances the plant resistance to Verticillium wilt [29]. However, it is barely reported that pectin is involved in plant resistance to powdery mildew.

Powdery mildew is one of the most widespread plant diseases caused by biotrophic fungi; it also suppressed plant immunity by secreting a series of effectors. For example, barley powdery mildew effector CSEP0055 interacts with the host protein PR17c in secondary penetration events [30]. Powdery mildew effector CSEP0162 targets barley endosomal MONENSIN SENSITIVITY1 to regulate plant immunity [31]. Powdery mildew protein GcR8IP1 interacts with RPW8.2 and amplifies its expression to boost immunity in *Arabidopsis* [32]. It was also identified that the powdery mildew effector BEC1054 candidate host targets [33].

Grapevine is an economic horticultural plant widely cultivated in the world [34]. Its fruit quantity and quality can be severely damaged by *Erysiphe necator* infection [35–37]. As an obligate biotroph fungi, the *E. necator* can release an arsenal of effectors from haustoria to suppress the grapevine immunity system and acquire nutrients from the host [38, 39]. However, studies on the molecular mechanism of *CSEPs* in *E. necator* have been barely clarified. In our previous studies, we published the genome of *En*. NAFU1, which is a virulence grapevine powdery mildew isolate in China, and the *CSEPs* have been predicted in the *En*. NAFU1 genome. We also found the effector CSEP087 interacts with VviADC to regulate grapevine immunity [40], but the molecular mechanisms of most effectors during *En*. NAFU1 infection are still largely unknown.

To explore the effectors playing functions during the *E. necator* infection to grapevine, in the present study, we characterized an effector CSEP080, which was located in plant chloroplasts and plasma membrane. CSEP080 was found to be a necessary effector for the *E. necator* pathogen. At the same time, *CSEP080* influences plant photosynthesis and inhibits INF1-elicited PCD in tobacco leaves. The CSEP080 interacted with the grapevine cytochrome b6-f complex iron–sulfur subunit VviB6f in chloroplast, and the grapevine pectinesterase VviPE in the plasma membrane. It also promoted the accumulation of VviB6f and VviPE. The *VviB6f* was proven to have impacts on the plant photosynthesis, which also negatively regulated grapevine hydrogen peroxide accumulation and plant resistance. Besides, the *VviPE* negatively regulated grapevine resistance to powdery mildew. Overall, our results show that, during the infection of *E. necator*, an effector CSEP080 is translocated into plant chloroplast and plasma membrane to influence plant photosynthesis, ROS production, pectin degradation and plant immunity, which indicated an effector of *E. necator* interferes with plant physiology to benefit infection.

## Results

### CSEP080 translocated to plasma membrane and chloroplasts that promotes plant photosynthesis

**Figure 1:**
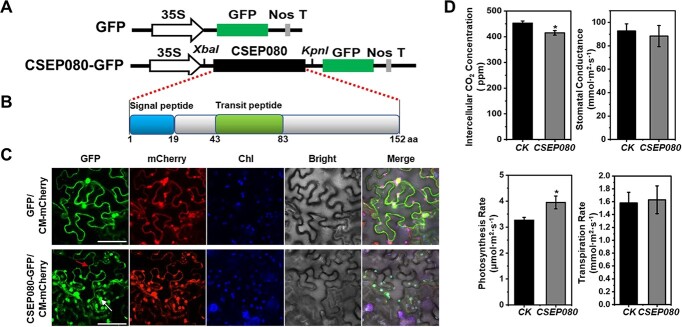
The plant photosynthesis capacity after the translocating of CSEP080 into the plasma membrane and chloroplasts. **A**, Vector illustrations of GFP and CSEP080-GFP. **B**, The signal peptide (1–19 aa) and chloroplast transit peptide (43–83 aa) in the CSEP080 predicted by SignalP 4.1 and LOCALIZER. **C**, The CSEP080 in the plant chloroplasts and plasma membrane. The fluorescence signals were examined by confocal microscopy. The red arrow represents the plasma membrane and the white arrow represents the plant chloroplast. Chl, chlorophyll. Bar = 50 μm. **D**, The photosynthesis traits of *N. benthamiana* affected by the *CSEP080*. *CK* means that leaves of *N. benthamiana* were transformed by *Agrobacterium* that carries the empty vector. The asterisks represent significant differences. (*P < 0.05).

To explore whether the effector translocated to plant chloroplasts in the interaction between powdery mildew and grapevine, the subcellular location of CSEPs were predicted by using the software LOCALIZER. Among the *E. necator* encoding CSEPs, the CSEP080 was predicted to be translocated in host chloroplasts and 43–83 amino acid is the chloroplast transit peptide (Fig. S1A and B, see online supplementary material). To determine whether the CSEP080 was translocated into plant chloroplasts, the vector of CSEP080-GFP was constructed and expresses fusion protein in tobacco leaves. The fluorescence signals of the CSEP080-GFP and CM-mCherry were co-localized and enriched in chloroplasts and plasma membrane in tobacco leaf epidermal cells under a confocal microscope (Fig. 1C). These results indicated that the CSEP080 was translocated into plant chloroplasts and plasma membrane.

The *CSEP080* was transient expressed in *Nicotiana benthamiana* leaves, and the plant photosynthetic rate was measured to determine if the *CSEP080* could impact photosynthesis. The intercellular CO_2_ concentration was decreased and the photosynthesis rate was improved when the plant expressed *CSEP080* (Fig. 1D). These results suggested that *CSEP080* did influence the plant photosynthesis.

### CSEP080 is a secreted effector of *E. necator*

**Figure 2:**
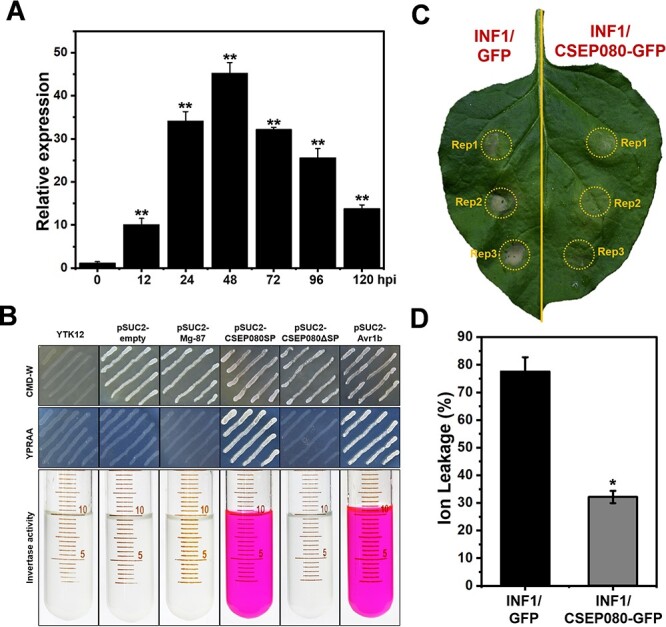
The expression pattern, secretion and suppressed PCD of CSEP080. **A**, Relative expression level of *CSEP080* assayed by RT-qPCR. **B**, Secreted validation of the CSEP080 by yeast system. **C**, CSEP080 suppressed INF1-induced PCD in *N. benthamiana*. **D**, Statistical analysis of the ion leakage of the *N. benthamiana*. (*P < 0.05, **P < 0.01).

To examine whether the CSEP080 has the characteristics as a powdery mildew effector, the expression pattern and secretion function of CSEP080 were examined. As shown in Fig. 2A, *CSEP080* was up-induced at 12–48 hours post inoculation (hpi) through RT-qPCR analysis, which was the key period that powdery mildew forms haustorium to secrete effectors into grapevine cells. In addition, CSEP080 was a 152-amino acid protein, which carried a 23 amino acid secreted signal peptide predicted by SignalP4.1 (Fig. S1C, see online supplementary material). To confirm the secretory function of CSEP080, a yeast strain YTK12, which was in lack of secreted invertase, a transformed CSEP080SP (the signal peptide of CSEP080), CSEP080ΔSP (deleted the signal peptide of CSEP080) and the Avr1bSP (the signal peptide of Avr1b) were grown on CMD-W and YPRAA medium. The yeast expressing CSEP080SP, which was similar to the positive control Avr1bSP, could grow on YPRAA medium, but the yeast expressing CSEP080ΔSP and the negative control failed to grow. Meanwhile, the invertase enzyme activity of yeast was detected by 2, 3, 5-triphenyltetrazolium chloride (TTC) (Fig. 2B). These results suggested that CSEP080 was an induced expression and secreted protein during the infection of powdery mildew.

Then, whether the *CSEP080* could suppress the plant hypersensitivity response, including PCD was examined. INF1 is an elicitor that could induce PCD in *N. benthamiana* leaves as reported previously [7]. As Fig. 2C and D shows, the CSEP080 could suppress plant PCD and decrease ion leakage when *N. benthamiana* leaves co-expressed CSEP080 and INF1.

**Figure 3:**
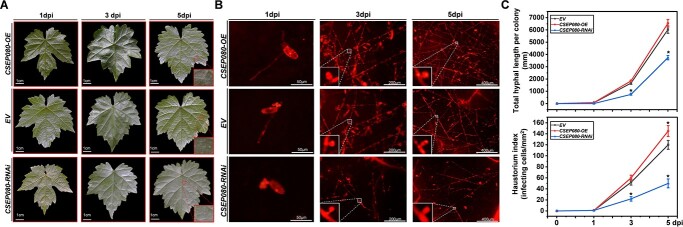
The necessity of *CSEP080* for the full virulence of *En.* NAFU1 in grapevine. **A**–**B**, The inhibition of the growth of powdery mildew mycelium in grapevine leaves after the silencing of *CSEP080*. **C**, Statistical analysis of total hyphal length per colony and haustorium index on the grapevine leaves transiently overexpressed and silenced *CSEP080* at 1, 3, and 5 dpi. (*P < 0.05).

To evaluate the *CSEP080* contribution in the *E. necator* pathogenicity, the *CSEP080* was transient overexpressed and silenced in the grapevine leaves by *agro*-infiltration, and then the *En*. NAFU1 spores were inoculated. The powdery mildew hyphae length per colony and haustorium index decreased on grapevine leaves when the *CSEP080* was silenced, compared to the grapevine leaves that were transformed empty vector and overexpressed *CSEP080* (Fig. 3). These results demonstrated that the CSEP080 was an indispensable effector for *E. necator* pathogenic.

### CSEP080 interacts with VviB6f and VviPE

**Figure 4:**
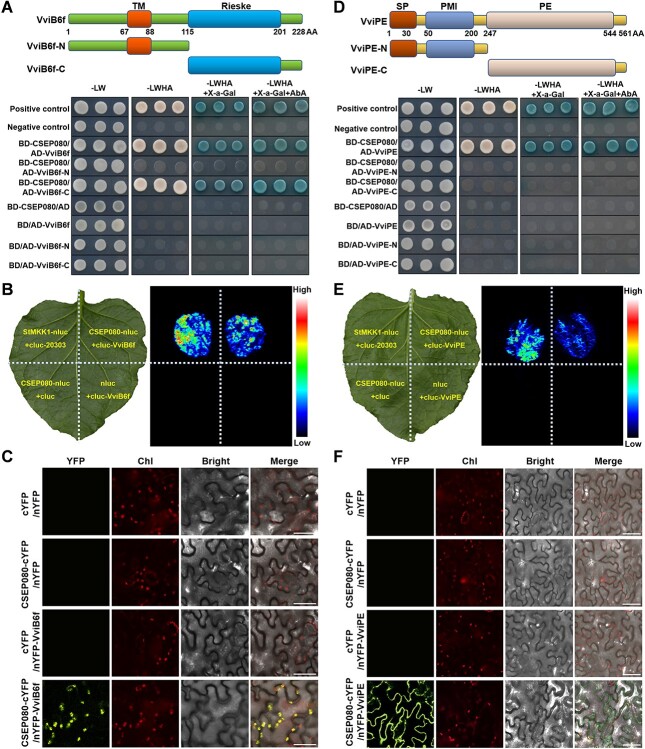
The interaction of the CSEP080 with the VviB6f and VviPE. **A**, Yeast two-hybrid assays detecting the interaction between the CSEP080 and the C-terminal of VviB6f. **B**, Luciferase complementation image verifying the interaction between the CSEP080 and the VviB6f. **C**, BiFC assays for the interaction between CSEP080 and VviB6f. Scale bar = 50 μm. **D**, Yeast two-hybrid assays detecting the interaction between the CSEP080 and the VviPE. **E**, Luciferase complementation image verifying the interaction between the CSEP080 and the VviPE. **F**, BiFC assays for the interaction between the CSEP080 and the VviPE. Scale bar = 50 μm.

To study the molecular mechanism of *CSEP080* during powdery mildew infection, a yeast two-hybrid (Y2H) library acquired from *E. necator*-infected grapevine leaves was constructed, and the screening of its host targets was performed using the CSEP080 as bait. A total of 42 candidate target proteins in grapevine were screened out (Fig. S2, TableS1, see online supplementary material). As CSEP080 was located in plant chloroplasts and plasma membrane, the VviB6f (grapevine cytochrome b6-f complex iron–sulfur subunit) and the VviPE (grapevine pectinesterase) were the focus which were predicted to locate in the chloroplasts and the plasma membrane, respectively. To confirm the interaction between CSEP080 and VviB6f, as well as the interaction between CSEP080 and VviPE, the *CSEP080* was cloned and recombined into the vector pGBKT7. The *VviB6f* and *VviPE* were also cloned, then recombined into the vector pGADT7. The yeast strain Y2HGold co-transformed with *CSEP080* and *VviB6f*, or *CSEP080* and *VviPE* were grown on the mediums SD-LW, SD-LWHA, SD-LWHA+X-α-gal and SD-LWHA+X-α-gal+AbA (100 mM Aureobasidin A), which performed like the positive control (Fig. 4A and D). To confirm their interaction *in vivo*, the *CSEP080* was inserted into vector nLUC, while the *VviB6f* and *VviPE* were inserted into vector cLUC. Then, CSEP080-nluc, cluc-VviB6f, and cluc-VviPE were expressed in *N. benthamiana* leaves through *Agrobacterium*-mediated gene expression. The *N. benthamiana* leaves that co-expressed CSEP080-nluc and cluc-VviB6f, CSEP080-nluc and cluc-VviPE showed fluorescence through luciferase complementation imaging, which is like StMKK1-nluc and cluc-20 303 reported previously (Fig. 4B and E) [41]. To further confirm their interaction, the *CSEP080* was inserted into vector pSPY-cYFP, and the *VviB6f* and *VviPE* were inserted into vector pSPY-nYFP. The *N. benthamiana* leaves that co-express CSEP080-cYFP and nYFP-VviB6f had fluorescence signals in chloroplasts, while the leaves co-expressing CSEP080-cYFP and nYFP-VviPE had fluorescence signals in plasma membrane under the confocal microscope, respectively. In contrast, the fluorescence signals in the control group were not observed (Fig. 4C and F; Fig. S3, see online supplementary material) [42]. To determine whether the CSEP080 interacted with the VviB6f and the VviPE regions, the domains of VviB6f and VviPE were analysed through software SMART. It turned out that VviB6f had two domains, transmembrane (TM) (67–88 AA) and Rieske (115–201 AA). VviPE had three domains, signal peptide (SP) (1–30 AA), plant pectin methylesterase inhibitor (PMI) (50–200 AA) and pectinesterase (PE) (247–544 AA). As shown in Fig. 4A, CSEP080 interacted with the C-terminal of VviB6f that contains the Rieske domain. However, CSEP080 didn’t interact with the N-terminal or C-terminal of VviPE through yeast two-hybrid (Fig. 4D). These results suggested that the CSEP080 did interact with the VviB6f and the VviPE.

### CSEP080 promotes VviB6f and VviPE accumulation

**Figure 5:**
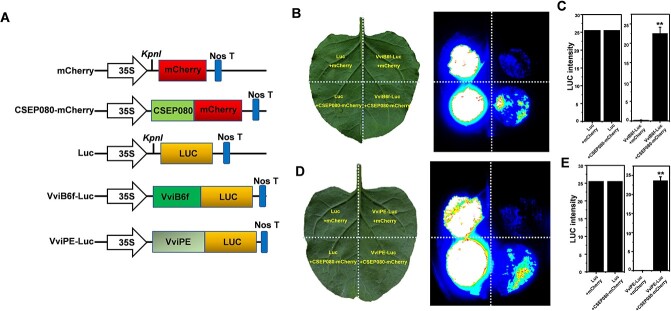
The accumulation promotion of the VviB6f and VviPE brought by the CSEP080. **A**, Vector illustrations of mCherry, CSEP080-mCherry, Luc, VviB6f-Luc, and VviPE-Luc. **B**, The promotion of the VviB6f accumulation brought by the CSEP080. **C**, Statistical analysis of LUC intensity in *N. benthamiana* leaves co-expressing CSEP080 and VviB6f. **D**, The promotion of the VviPE accumulation brought by CSEP080. (^**^P < 0.01).

Because CSEP080 could interact with VviB6f and VviPE, we wondered if CSEP080 could promote VviB6f and VviPE accumulation or degradation. Based on this hypothesis, vectors of CSEP080-mCherry, VviB6f-Luc and VviPE-Luc were constructed and transient co-expressed in *N. benthamiana* leaves. The fluorescence signals of the expressing of Luc + mCherry was similar to the expressing of Luc + CSEP080-mCherry, but the LUC intensity of them was not different, which meant the CSEP080 did not regulate the Luc accumulation or degradation. However, the fluorescence signals from VviB6f-Luc + CSEP080-mCherry expressing were lighter than that of VviB6f-Luc + mCherry expressing, similar to the difference between the VviPE-Luc + CSEP080-mCherry and VviB6f-Luc + mCherry. Meanwhile, the statistical results of LUC intensity analysis were the same as the observation (Fig. 5), so the data demonstrated the promotion of CSEP080 to VviB6f and VviPE accumulation.

### VviB6f is a chloroplast protein that promotes plant photosynthesis

**Figure 6:**
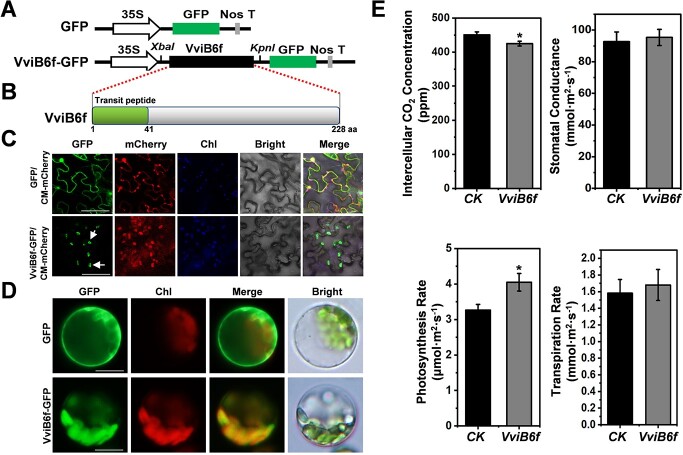
The VviB6f located in plant chloroplasts regulating plant photosynthesis. **A**, The vector illustrations of GFP and VviB6f-GFP. **B**, The prediction of the chloroplast transit peptide (1–41 aa) in the VviB6f. **C**, The locating of the VviB6f in the *N. benthamianat* chloroplasts. Chl, chlorophyll. Bar = 50 μm. **D**, The VviB6f localizing in the chloroplasts of the grapevine protoplasts. **E**, The *N. benthamiana* photosynthesis traits influenced by *VviB6f*. *CK* means that leaves of *N. benthamiana* were transformed by *Agrobacterium* that carries empty vector. (^*^P < 0.05).

The interaction of CSEP080 and VviB6f in plant chloroplasts was shown in Fig. 4C, and B6f is a cytochrome b6-f complex iron–sulfur subunit that plays a role in plant photosynthesis. Firstly, we analysed the VviB6f location and constructed phylogenetic tree. As shown in Fig. S4A (see online supplementary material), the *VviB6f* was located in Chromosome 19 (4382462-4 384 537 bp). Meanwhile, we found most plants belonging to the same genera were grouped into the same cluster according to the B6f phylogenetic tree. The C-terminal of B6f sequences containing the Rieske domain was conservative, but the N-terminal containing the TM domain was less conservative (Fig. S4B, see online supplementary material). Secondly, the VviB6f (XP_034679439.1) was a 228-amino acid protein, which predicted that the 1–41 amino acid transited was chloroplast peptide through LOCALIZER (Fig. 6A and B; Fig. S5, see online supplementary material). To check the location of VviB6f in the plant cell, we constructed the vector VviB6f-GFP and transient expressed the fusion protein in *N. benthamiana* leaves. As shown in Fig. 6C, the fluorescence signals were enriched in plant chloroplasts under a confocal microscope. Meanwhile, the VviB6f-GFP transient expressed in grapevine protoplasts also had the fluorescence signals observed in chloroplasts (Fig. 6D). To determine whether the *VviB6f* affected plant photosynthesis, we transient expressed the *VviB6f* in *N. benthamiana* leaves and measured the plant photosynthesis. The intercellular CO_2_ concentration decreased and the photosynthesis rate improved under the expression of *VviB6f* (Fig. 6E). These results suggested that the VviB6f was located in the plant chloroplasts and influenced plant photosynthesis.

### *VviB6f* negatively regulates grapevine resistance to *E. necator* and hydrogen peroxide accumulation

**Figure 7:**
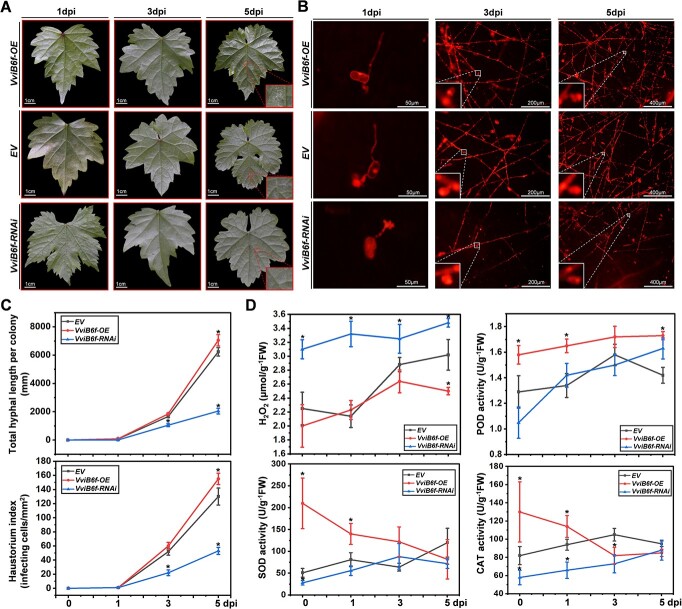
The *VviB6f* regulating the grapevine resistance to powdery mildew. **A**–**B**, Silencing *VviB6f* inhibits the growth of powdery mildew mycelium on grapevine leaves. **C**, Statistical analysis of total hyphal length per colony and haustorium index on the grapevine leaves transiently overexpressed and silenced *VviB6f* at 1, 3, and 5 dpi. **D**, Hydrogen peroxide content and ROS scavenging activities measured in grapevine leaves that transiently overexpressed and silenced *VviB6f* during powdery mildew infection. (^*^P < 0.05).

To study the function of *VviB6f* in regulating the grapevine resistance to *E. necator*, we transiently overexpressed and silenced *VviB6f* in grapevine leaves by *agro*-infiltration grapevine leaves, and then inoculated the *En*. NAFU1 spores. The powdery mildew hyphae length and haustorium were observed by propidium iodide staining at 1, 3, and 5 dpi. The powdery mildew hyphae length reduced and the haustorium index decreased on the grapevine leaves which were transient silenced the *VviB6f*, as compared with the grapevine leaves that transformed the empty vector and overexpressed *VviB6f* (Fig. 7A–C). These results indicated that the *VviB6f* negatively regulated the grapevine resistance to *E. necator*.

B6f connects PSII and PSI of the photosynthetic electron transport chain, which affects the CO_2_ assimilation rate and splits water molecules into photon and molecule oxygen (Fig. S6, see online supplementary material) [18, 43]. Hence, we transient overexpressed and silenced the *VviB6f* in grapevine leaves and measured the leaf hydrogen peroxide concentration, and the activity of catalase (CAT), peroxidase (POD), and superoxide dismutase (SOD) during *E. necator* infection. The hydrogen peroxide concentration of the grapevine leaves that transient silenced the *VviB6f* was higher than those that transformed the empty vector and overexpressed the *VviB6f* at 0, 1, 3, and 5 dpi. Similarly, the activity of CAT, POD, and SOD in grapevine leaves that transiently overexpressed the *VviB6f* were higher than those that transformed the empty vector and silenced *VviB6f* at 0 and 1 dpi (Fig. 7D). These data suggested that *VviB6f* negatively regulates grapevine hydrogen peroxide accumulation.

### *VviPE* negatively regulates grapevine resistance

**Figure 8:**
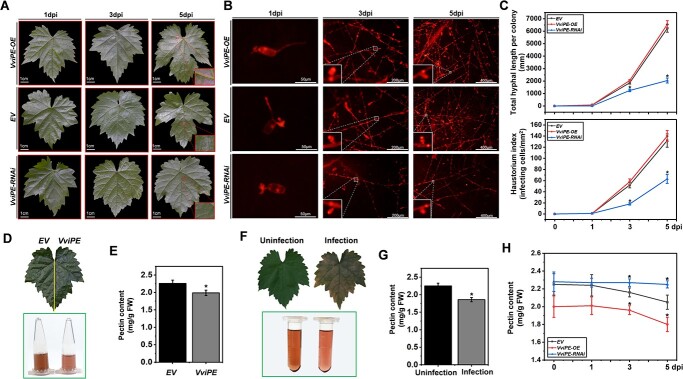
The resistance of the grapevine to the powdery mildew weakened by the *VviPE*. **A**–**B**, The inhibition of the growth of powdery mildew mycelium in grapevine leaves after the silencing of *VviPE*. **C**, Statistical analysis of total hyphal length per colony and haustorium index on the grapevine leaves transiently overexpressed and silenced *VviPE* at 1, 3, and 5 dpi. **D**, Extracting pectin content in grapevine leaves that was transformed empty vector and *VviPE*. **E**, Statistical analysis of pectin content in grapevine leave that was transformed empty vector and *VviPE*. **F**, Extracting pectin content in grapevine leaves. G, Statistical analysis of pectin content in powdery mildew-infected and uninfected grapevine leaves. **H**, Statistical analysis of the pectin content in the *VviPE* overexpressing and gene silencing grapevine leaves during *E. necator* infection. (^*^P < 0.05).

Because *E. necator* effector CSEP080 manipulated host VviPE as shown in Fig. 4, we wanted to know if *VviPE* played a role in the interaction between grapevine and *E. necator*. Therefore, the *VviPE* location was analysed and a phylogenetic tree was constructed. The *VviPE* was located in Chromosome 17 (3365207-3 368 241 bp). The phylogenetic tree showed that the location of VviPE was similar to the model plant *Arabidopsis thaliana* (Fig. S7, see online supplementary material). In addition, we transiently overexpressed and silenced *VviPE* in grapevine leaves by *agro*-infiltration and inoculated the *En*. NAFU1 spores. As Fig. 8A–C shows, the powdery mildew hypha and haustorium index decreased when the *VviPE* was transient silenced in grapevine leaves at 3, 5 dpi, compared with the grapevine leaves transforming empty vector and overexpressing *VviPE*. VviPE has pectinesterase domain, so we wanted to explore whether VviPE has the function of pectinesterase in grapevine. The grapevine leaf transformed the empty vector and the *VviPE* by *agro*-infiltration, and measured the pectin content. The pectin content of the grapevine leaves expressed *VviPE* lower than the grapevine leaf transformed empty vector (Fig. 8D and E). These results indicated that *VviPE* negatively regulated grapevine resistance to *E. necator* and had a function in promoting pectin degradation.

As *E. necator* effector CSEP080 interacts with VviPE and *VviPE* negatively regulating grapevine resistance, we wanted to know whether pectin content is reduced during powdery mildew infection. The pectin concentration of grapevine leaves which were infected or not infected by *E. necator* were then measured. As shown in Fig. 8F and G, the pectin content of grapevine leaves infected by *E. necator* was lower than that of uninfected leaves. In addition, the pectin content in the grapevine leaves transiently overexpressing the *VviPE* was lower than those that transformed the empty vector and silenced the *VviPE* at 0, 1, 3, and 5 dpi (Fig. 8H). These results indicated that *E. necator* infection will degrade grapevine leaves’ pectin content.

## Discussion

In the interaction between pathogens and plants, pathogens secrete an arsenal of effectors into plant cells to manipulate host targets, which modulates plant immunity and is beneficial to infection [44]. Recently, scientists reported molecular mechanisms of effectors during pathogens infection. For example, the RXLR effector PITG20303 from *P. infestans* interacts with the host MKK1 protein to regulate plant immunity [41]. The fungal *Magnaporthe oryzae* effector AvrPiz-t structurally mimics Ca2^+^-sensor, RESISTANCE OF RICE TO DISEASES1 (ROD1) activates the same ROS-scavenging cascade to suppress host immunity and to promote virulence [45]. In this study, an effector of *E. necator*, CSEP080, was found be able to translocate into host chloroplasts and plant plasma membrane, which could suppress the grapevine immunity system by targeting chloroplast protein VviB6f to retard the synthesis of hydrogen peroxide, and manipulating the VviPE to promote pectin degradation.

Chloroplasts play a crucial role in activating plant defensive hormonal responses during plant–pathogen interactions [15]. It is also a place for ROS production and primary metabolisms, which act as defense signals for plant immunity [18]. Plant chloroplasts interconnect haustoria to form dynamic organelle clusters when the Irish potato famine pathogen *P. infestans* infects the host [22]. Pathogens secrete virulence factors that target chloroplasts and regulate the production of defense molecules. For example, a haustorium-secreted effector (Pst_12806) from *Pst* translocated into chloroplasts, interacting with the C-terminal Rieske domain of the wheat chloroplasts protein TaISP to inhibit PCD. In addition, the wheat TaISP protein also interacts with two low sequence similarity effectors, which suppresses the host ROS accumulation and promotes the fungal pathogenicity [24, 25]. In the interaction between *P. viticola* and grapevine, a candidate *P. viticola* effector RXLR31154 would interact with *V. piasezkii* chloroplast protein VpPsbP to reduce H_2_O_2_ accumulation, and activate the ^1^O_2_ signaling pathway, benefitting the *P. viticola* infection [23]. These findings agreed with our experiment. The effector CSEP080 secreted from *E. necator,* which was translocated in plant chloroplasts and plasma membrane, interacting with grapevine chloroplast protein VviB6f, could affect plant photosynthesis and negatively regulate the grapevine hydrogen peroxide production and plant resistance to powdery mildew (Figs 1 and4–6).

Pectin plays a vital role in constitutive and inducible defenses [27]. Pectin lyase regulates cell apoptosis of *V. dahliae*, which results in plant resistance to Verticillium wilt [28]. In *A. thaliana*, the pectin biosynthesis was impaired, which made the pathogen easier to penetrate the epidermis [27]. In this study, CSEP080 interacts with VviPE to promote pectin degradation (Figs 4 and8). Hence, pectin plays a vital role in regulating plant resistance to pathogens.

Grape quantity and quality was ruined by a destructive pathogen powdery mildew [46, 47]. Although many pathogen-related genes have been cloned from resistant grapevine in recent years, the molecular mechanisms of plant resistance to powdery mildew have been little revealed [48–50]. However, because of the restriction of reliable transformation systems for *E. necator* and the genetic transformation of grapevine being time-consuming work [38, 51], few reports clarified powdery mildew pathogenesis. In addition, we published the virulence grapevine powdery mildew isolate *En*. NAFU1 genome and predicted the CSEPs encoded in the *En*. NAFU1 genome recently. We reported that a secreted effector CSEP087 was encoded in the *En*. NAFU1 targets VviADC, which is an arginine decarboxylase to interfere with grapevine immunity [40]. The molecular mechanisms of most effectors in *En*. NAFU1 infection are still largely unknown. In this research, we identified a suppressing plant hypersensitivity response effector CSEP080 from *En*. NAFU1 and explored its molecular mechanisms in *En*. NAFU1 virulence (Figs 2 and3).

**Figure 9:**
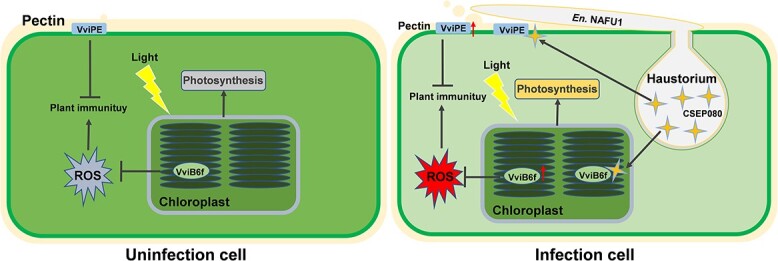
The working model of the *CSEP080* in the *En.* NAFU1–grapevine interaction. In uninfected grapevine cells lacking CSEP080, VviB6f plays function normally in photosynthesis, keeping ROS and pectin at a normal level. In powdery mildew infected grapevine cell, an effector CSEP080 was secreted from *En*. NAFU1 haustorium and translocated into grapevine cell chloroplasts and plasma membrane. In chloroplasts, CSEP080 interacts with VviB6f to improve plant photosynthesis rate and inhibit ROS accumulation, which suppresses plant immunity. In the plasma membrane, CSEP080 manipulates VviPE to degrade plant pectin, which is beneficial to *En*. NAFU1 infection.

In conclusion, we identified a translocated plant chloroplast and plasma membrane *E. necator* effector, CSEP080, which was induced in powdery mildew infection and suppressed plant hypersensitivity response. The CSEP080 is an indispensable effector for *E. necator* pathogenetics, and the *E. necator* virulence was decreased when *CSEP080* was silenced. Moreover, CSEP080 could interact with grapevine chloroplast protein VviB6f, affecting the plant photosynthesis and negatively regulating grapevine hydrogen peroxide production and plant resistance. Meanwhile, the CSEP080 could interact with VviPE to promote pectin degradation (Fig. 9). These results help to understand the effector function in the interaction between powdery mildew and grapevine and promote exploration in the molecular mechanism of grapevine powdery mildew effectors in the future.

## Materials and methods

### Plant and pathogen materials

*Vitis vinifera* cv. Thompson Seedless was used for explant establishment, which was planted in the Yangling, Shaanxi, People’s Republic of China. The grapevine was cultured in a tissue culture laboratory. After the tissue generation and redifferentiation, the grapevine was transplanted into the soil, and cultivated in an incubator under 23°C, (16 h light/8 h dark). The *Nicotiana benthamiana* plant used for protein expression and protein subcellular localization was cultivated in a greenhouse for 4 weeks from seedlings.

*En*. NAFU1 was identified in 2016 as reported previously [52]. The *En*. NAFU1 was maintained on the clean grapevine leaves of ‘Thompson Seedless’ in an incubator under 23°C conditions for 6 years. To assess the influence of *CSEP080* to powdery mildew pathogenic, as well as how the *VviB6f* and *VviPE* regulate the grapevine resistance to the *E. necator*, the fresh *En*. NAFU1 spores were collected from *V. vinifera* cv. Thompson Seedless leaves. Then, powdery mildew spores were inoculated on the grapevine leaves.

### Bioinformatics analysis

The signal peptide of CSEP080 was analysed through SignalP 4.1 (http://www.cbs.dtu.dk/services/SignalP-4.1/). To do multiple sequence alignment, we use the software ClustalX. To analyse the protein location, LOCALIZER (http://localizer.csiro.au/index.html) was used for predicting protein subcellular localization. In order to observe the gene expression changes in powdery mildew infection and clone the genes, the primers were designed with the Vector NTI. The MEGA6.0 was used in the sequence analysing gene evolution. Gene sequences were searched and downloaded in the NCBI database (https://www.ncbi.nlm.nih.gov/). To analyse the conservative domain in protein, the sequence was loaded in the SMART for analysis.

### Plasmid constructions

The vectors pSUC2-CSEP080∆SP and pSUC2-CSEP080SP were constructed by using the restriction sites *BamHI* and *EcoRI* to confirm the secretion function of CSEP080. To confirm the proteins CSEP080 and VviB6f subcellular localization in the *N. benthamiana* and grapevine protoplasts, cloning *CSEP080* and *VviB6f* fragments were recombined into the pCAMBIA2300 vector using *Kpn1* to form CSEP080-GFP and VviB6f-GFP. The regulating function of *CSEP080*, *VviB6f*, and *VviPE* in the powdery mildew virulence and grapevine resistance were also assayed, and the vectors used for grapevine leaves transient expression were CSEP080-GFP, VviB6f-GFP, and VviPE-GFP, which were constructed by recombining into the vector pCAMBIA2300 with the restriction site *Kpn1*. The silencing vector was the pK7WIWG2D, and the *CSEP080-RNAi*, *VviB6f-RNAi*, and *VviPE-RNAi* were built using the Invitrogen gateway recombinase (Thermo Fisher Inc, Carlsbad, CA, USA).

For assaying the protein interaction in yeast two-hybrid (Y2H), *CSEP080* was recombined into vector pGBKT7 using restriction sites *BamHI* and *EcoRI*. The *VviB6f* and *VviPE* were cloned from the cDNA of *V. vinifera*. cv Thompson Seedless, and then recombined into vector pGADT7 using restriction sites *BamHI* and *XhoI*. To identify the region of interaction between the CSEP080 and VviB6f, or between the CSEP080 and VviPE, the fragments of *VviB6f* and *VviPE* were ligated into pGADT7. For bimolecular fluorescence complementation (BiFC) assays, the *CSEP080*, *VviB6f*, and *VviPE* were cloned into pSPY-CE and pSPY-NE using the *Kpn1* site to form the CSEP080-cYFP, nYFP-VviB6f, and nYFP-VviPE plasmids, respectively. For assaying firefly luciferase complementation imaging (LCI), the *SEP080* was recombined into pCAMBIA-nLuc, and the *VviB6f* and *VviPE* were recombined into pCAMBIA-cLuc by using *Kpn1* and *Sal1* sites to form the CSEP080-nluc, cluc-VviB6f and cluc-VviPE plasmids, respectively. Also, the *VviB6f* and *VviPE* were recombined into the pCAMBIA2300-Luc by using *Kpn1* to form VviB6f-Luc and VviPE-Luc plasmids, respectively, to assay the regulating function of the CSEP080 to the VviB6f and VviPE. The primers used for plasmid construction are shown in Table S2.

### Secretion assay

To verify the secreting function of CSEP080, the yeast strain YTK12 containing the vectors pSUC2-CSEP080SP, pSUC2-CSEP080ΔSP, pSUC2-Avr1b, and pSUC2-Mg87 grown on YPRAA medium and 2, 3, 5-triphenyltetrazolium chloride (TTC) were used to assay the invertase activity. The method of yeast secretion system was described previously [10, 53, 54].

### *Agrobacterium*-mediated gene expression

*Agrobacterium tumefaciens* containing vector was cultured in liquid LB medium at 28°C for 8 hours. Then, the *A. tumefaciens* cells were centrifuged at 5000 rpm, 28°C, for 5 min. The bacteria were resuspended in a solution containing 10 mM MgCl_2_. Then, the grapevine leaves were infected with the prepared *A. tumefaciens* GV3101, then cultivated under 28°C conditions for 3 days [55].

*Agrobacterium*-mediated infiltration assays were performed according to the methods previously described [40]. The process of the bacteria injecting for tobacco leaves was similar to that for grapevine. For bimolecular fluorescence complementation and luciferase complementation imaging, the tobacco leaves were injected with diluted culture solution. After 3 days culturing time, the gene expression was confirmed by observing the fluorescent signal under a confocal microscope.

### Gene expression assay

To study the expression level of *CSEP080* during infecting of the powdery mildew to the grapevine, the ‘Thompson Seedless’ leaves were inoculated with *En*. NAFU1 spores. The samples were collected at 0, 12, 24, 48, 72, 96, and 120 hpi, then pre-cooled in liquid nitrogen and stored in a −80°C scientific refrigerator. The total RNA was extracted using a Quick RNA isolation kit (R6827, Omega, Norcross, Georgia, USA) according to the manufacturer’s instructions. The cDNA was synthesized by using the Prime Script RT reagent Kit (6110A, TaKaRa, Shiga, Japan). The cDNA used for RT-qPCR was diluted and added with the SYBR Green master mix (RR430S, TaKaRa, Shiga, Japan). To monitor the relative expression level of genes, the *EnEF1* was used as an internal reference gene for *En*. NAFU1. The relative expression levels of *CSEP080* were calculated using the comparative 2^–ΔΔCT^ method.

### Electrolyte leakage rates

The electrolyte leakage rates was measured following the procedures described previously [54]. Briefly, three tobacco leaf discs containing the expressed protein were immersed in the 0.4 M mannitol solution. The mixture was kept shaking for 3 hours at room temperature, and the initial EC was measured with a conductivity meter (ST3100C). Then, the mixtures were put in a 85°C water bath for 20 min, and the EC after heating was measured again. The electrolyte leakage rates were calculated as the initial and total conductivity percentages.

### Measurement of photosynthetic rate

In order to check whether *CSEP080* and *VviB6f* influenced plant photosynthesis, *CSEP080* and *VviB6f* were expressed in the tobacco leaves and plant photosynthetic rate was measured. The tobacco leaf tissues were injected by agrobacterium carrying *CSEP080* or *VviB6f,* and then resuspended by the prepared solution (10 mM MgCl_2_, 10 mM acetosyringone). The plant photosynthesis capacity parameters, including intercellular CO_2_ concentration, stomatal conductance, photosynthesis rate and transpiration rate, were measured after 3 days of the injection with the CIRAS-3 system (PPSYSTEMS, 718 USA), following the manufacturer’s instructions and software.

### Measurement of plant pectin

The grapevine leaves sample with or without the powdery mildew infection were ground in liquid nitrogen. The pre-cooling 75% ethanol was added into samples, staying for 20 min. Then, the samples were centrifuged at 10 000 *g* for 10 min. Then, the original precipitate was washed separately by four different agents, following the order of glacial acetone, glacial, chloroform, and methanol mixture (V:V = 1:1), and glacial methanol. Each washing took 20 minutes, and after each wash the mixture was re-centrifuged at 10 000 g for 10 minutes to discard the supernatant. The final precipitate was dried in an oven at 60°C, and extracted twice in a boiling water bath for 1 h, with a 0.1% NaBH4 (pH = 4) and 0.5% ammonium oxalate buffer. The pectin was tested in the supernatant. The 100 μL supernatant or standard sample was transferred to a 1.5 mL centrifuge tube and dissolved in 98% H_2_SO_4_ and 12 mM Na_2_B_4_O_7_·10H_2_O solution. The mixture was incubated in boiling water for 5 minutes, cooled on ice, added with 20 μL of C_6_H_5_C_6_H_4_OH, then stayed for 25 minutes at 25°C. The finished samples solutions measured an absorbance of 520 nm and calculated the pectin content based on the standard.

### Y2H assay

In order to screen the CSEP080 interacting proteins in the grapevine, as well as co-transform the CSEP080-BD and grapevine cDNA yeast library into yeast strain Y2Hgold, we scribbled the yeast on the SD/−Trp-Leu-His-Ade medium containing 100 mM Aureobasidin A (AbA), and staining with X-α-gal. After 3 days of 30°C incubation, the yeast was sequenced through Sanger sequencing. For the Y2H assay, the yeast strain Y2HGold co-transformed with the vectors CSEP080-BD and VviB6f-AD or CSEP080-BD and VviPE-AD were scribbled on SD/−Trp-Leu medium. The interactions were confirmed by the yeast grown on the SD/−Trp-Leu-His-Ade medium containing 100 mM AbA, and staining with X-α-gal. The yeast co-transformed with Lam-BD and AD was the negative control. The yeast co-transformed with P53-BD and AD, which was self-activation, was the positive control.

### Fluorescence observation

*A. tumefaciens* containing CSEP080-GFP and VviB6f-GFP was injected into tobacco leaves. After three days incubation, the protein CM-mCherry (a marker protein CPK16 on plant chloroplasts and plasma membrane [19]) was expressed and the fluorescence signals in the tobacco leaves were observed under a confocal microscope.

The bimolecular fluorescence complementation was tested following the procedures described previously [40]. To confirm the interactions between the CSEP080 and VviB6f, and between the CSEP080 and VviPE by the method of bimolecular fluorescence complementation, the *A. tumefaciens* containing vectors was shaken to 0.4–0.6 of OD_600_ overnight. The *N. benthamiana* leaves were injected with a mixture of diluted cultures which were a mixed mixture (V:V = 1:1). After three days of incubation, the fluorescence signals that emerged in the tobacco leaves were observed by using a confocal laser scanning microscope (LEICA TCS SP8, Germany).

### Grapevine protoplast system

The grapevine protoplast system was measured following the procedures described previously [56]. The collected grapevine leaves were hydrolyzed with cellulase and pectinase overnight. The grapevine protoplast was obtained by filtering with a filter. The grapevine protoplast was then used for expressing the protein.

### Luciferase complementation imaging

The transient expression of tobacco leaves was done following the procedures described previously [40]. The *N. benthamiana* leaves transiently expressed the proteins of nLuc, cLuc, CSEP080-nluc, cluc-VviB6f, and cluc-VviPE. After three days of injection, the tobacco leaves separated from the plants were smeared with the substrate in the dark for 5 minutes to observe the fluorescence intensity in a live presenting camera (Lumazone Pylon 2048B, Princeton), reflecting the protein interactions.

### Luciferase intensity analysis

The *N. benthamiana* leaves were injected by the agrobacterium (1:1) carrying different vectors, and then transiently expressed the Luc + mCherry, Luc + CSEP080-mCherry, VviB6f-Luc + mCherry, VviB6f-Luc + CSEP080-mCherry, VviPE-Luc + mCherry, and VviPE-Luc + CSEP080-mCherry. The method for measuring the fluorescence signals was same as luciferase complementation imaging. The luciferase intensity was analysed by the software ImageJ (National Institutes of Health) that chose five typical regions.

### Confocal microscopy

The fluorescence in the leaves of grapevine and tobacco after *Agrobacterium* infected were observed through TCS SP8 SR (Leica, Germany). For BiFC and protein subcellular localization assays, the wavelength of the GFP laser was 488 nm, and the detection wavelength was 500–540 nm. For observing the mCherry, the detection wavelength was 570–620 nm, and the laser wavelength was 561 nm. To exclude the autofluorescence of chloroplasts, the laser wavelength was 640 nm, and the detection wavelength was 650–750 nm. All confocal images were conducted with software Olympus Fluoview. The pictures were acquired by the software ImageJ (National Institutes of Health).

### Evaluation of grapevine resistance to powdery mildew

For the assay of the grapevine resistance to powdery mildew, the grapevine leaves that transient transformed with *Agrobacterium* were inoculated with grapevine powdery mildew *En*. NAFU1 spores. The grapevine leaves were injected by the *Agrobacterium* carrying vectors, and the clean *En*. NAFU1 spores was inoculated after 2 days of injection. The plants were cultured in an incubator at 23°C under a 16 h light/8 h dark cycle. The hyphae growth status of *En*. NAFU1 on the grapevine leaves was stained by propidium iodide (PI) at 1, 3, and 5 dpi, observing with a microscope (Olympus BX51: Olympus Corp., Tokyo, Japan).

### Hydrogen peroxide assay

To test whether the chloroplast protein VviB6f affected plant hydrogen peroxide content and ROS scavenging enzyme in the grapevine, we used a transformation method to transiently express the *VviB6f* in grapevine leaves, and detected the hydrogen peroxide content. Hydrogen peroxide and ROS scavenging enzyme assay was performed according to the methods described previously [40].

### Statistical analysis

The experiment was conducted with three biological repetitions. The treatment effect was detected through ANOVA, performed in IBM SPSS v26.0.0 (IBM), the figures were plotted through Origin 2021 (originlab). The statistical significance was tested through Student’s *t*-test that was performed by GraphPad Prism 5 (https://www.graphpad.com/scientificsoftware/prism/). Significant differences are indicated with asterisks: * for *P* < 0.05 and ** for *P* < 0.01.

## Supplementary Material

Web_Material_uhad163

## Data Availability

All relevant data in this study are provided in the article and its supplementary data.
