# Supplementary material for: An effector of Erysiphe necator translocates to chloroplasts and plasma membrane to suppress host immunity in grapevine
Source: Hortic Res. 2023 Aug 16;10(9):uhad163. doi: 10.1093/hr/uhad163 (PMC10516348; doi:10.1093/hr/uhad163)
Supplement: Web_Material_uhad163 [file web_material_uhad163.pdf]

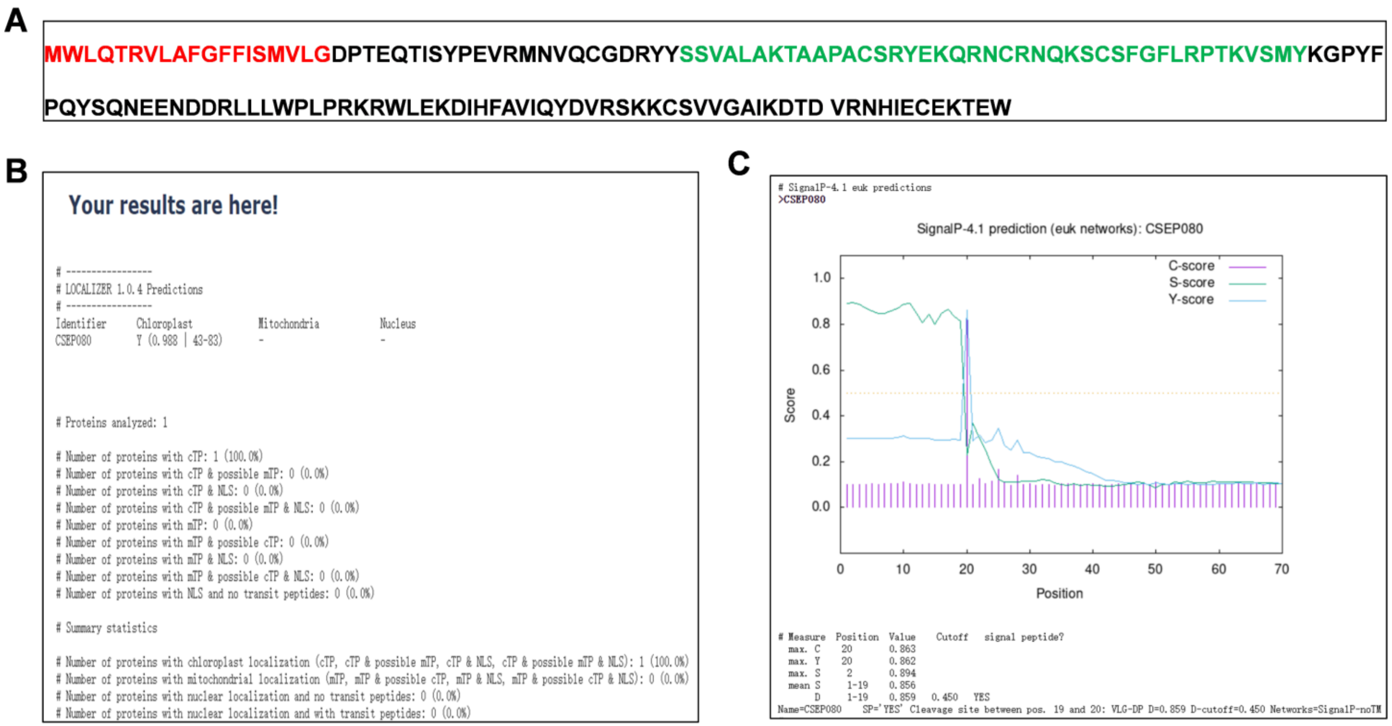

Supplemental Figure 1. CSEP080 is predicted to be secreted in plant chloroplasts. A, The protein sequence of CSEP080. Red represents the signal peptide and the green represents chloroplast transit peptide. B, The grapevine powdery mildew effector CSEP080 has a chloroplast transit peptide. It shows that amino acids 43–83 is chloroplast transit peptide after the amino acid sequence of CSEP080 is input into the software LOCALIZER. C, The grapevine powdery mildew effector CSEP080 has a signal peptide. It shows that amino acids 1-19 is signal peptide after the amino acid sequence of CSEP080 input into the software Signal4.1.

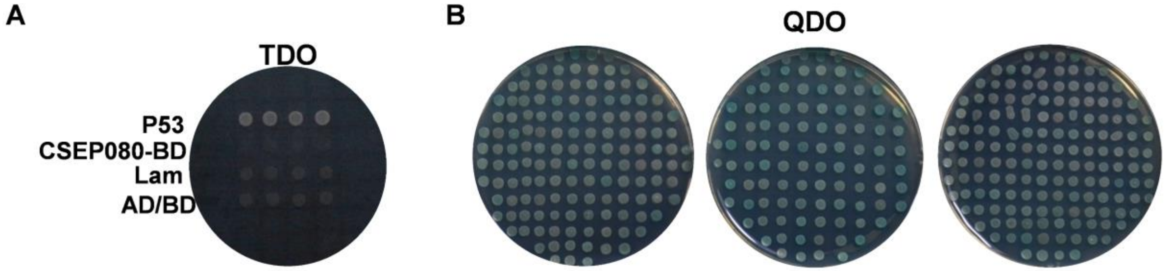

Supplemental Figure 2. CSEP080 transcriptional activation analysis and screens interacting proteins by yeast two-hybrid. A, Transcriptional activation analysis of CSEP080 in yeast. Growth of yeast containing CSEP080 on SD/-Ade/-Leu/-Trp medium. B, Screening of CSEP080 interacting proteins by yeast two-hybrid. Growth of yeast on SD/-Ade/-His/-Leu/-Trp medium.

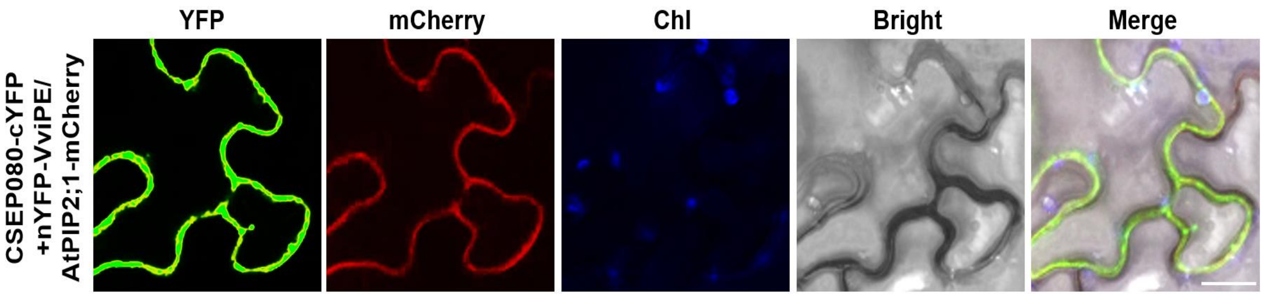

Supplemental Figure 3. CSEP080 interacts with VviPE on plasma membrane. AtPIP2;1 is a marker protein onplasma membrane. Scale bar=20µm.

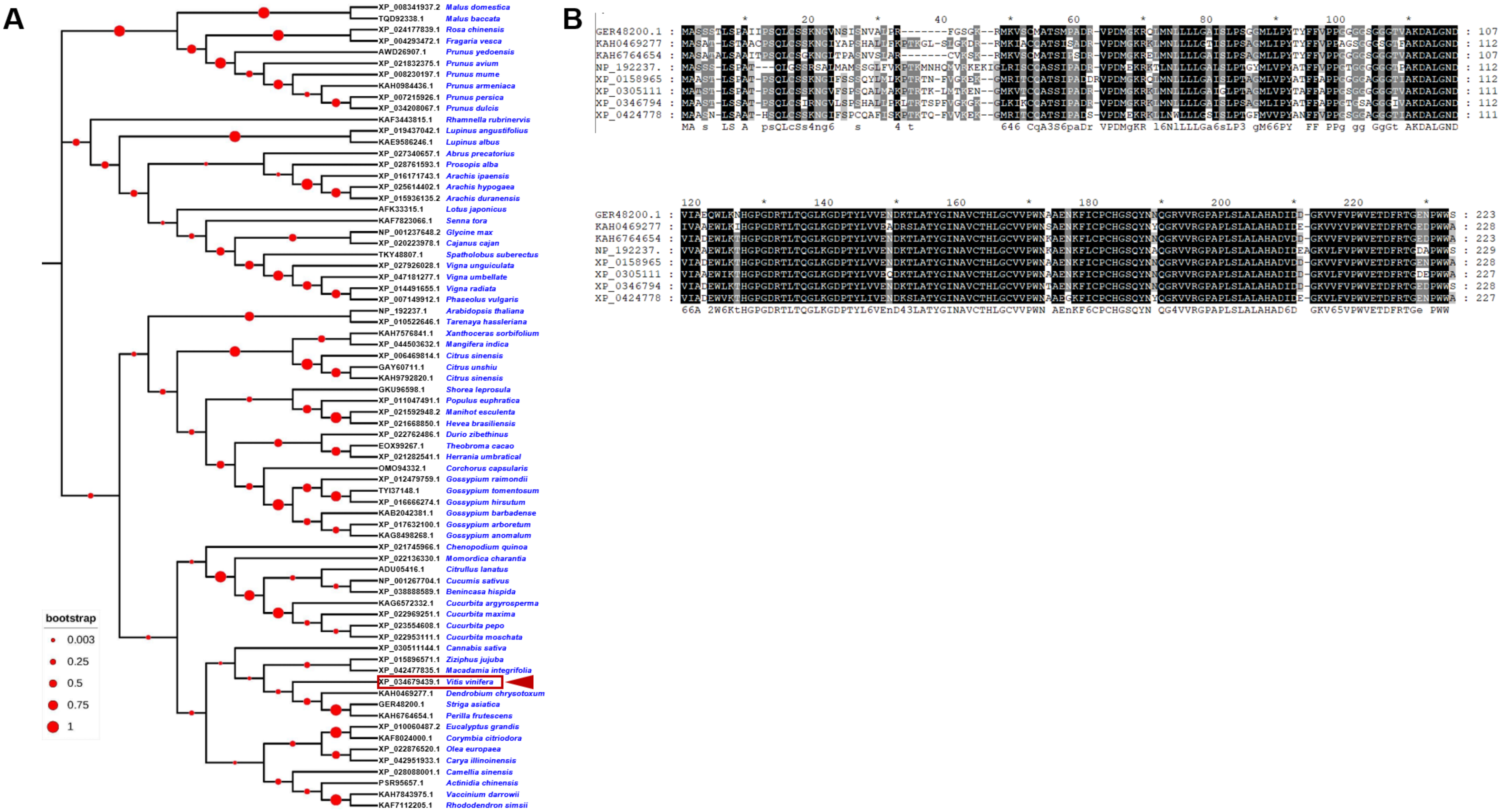

Supplemental Figure 4. The analysis of VviB6f. A, Phylogenetic analysis of VviB6f. Phylogenetic analysis with horticultural plants and model plant *Arabidopsis thaliana*, which was carried out by the MEGA6 software using the maximum likelihood method. The red triangle points to VviB6f. B, Sequence analysis of VviB6f. The VviB6f amino acid sequences were alignment to plants that clustered in the same cluster downloaded from NCBI.

A

MAASTLSSATPSQLCSIRNGVLSPSHALLPKLTRTSPFVGKGKGLKIKCQATSIPADRVPDMGKR

KLMNLLLLGAISLPSAGMLIPYATFFAPPGTGSAGGGIVAKDALGNDVIADEWLKTHGPGDRTLT

QGLKGDPTYLVVENDKTLATYGINAVCTHLGCVVPWNTAENKFICPCHGSQYNNQGRVVRGPAP

LSLALAHADIDDGKVV FVPWVETDFRTGEDPWWS

B

Your results are here!

```
# -----------------
# LOCALIZER 1.0.4 Predictions
# -----------------
Identifier                                                        Chloroplast          Mitochondria          Nucleus
XP_034679439.1 cytochrome b6-f complex iron-sulfur subunit,
chloroplastic-like [Vitis riparia]                                Y (1.0 | 1-41)       -                     Y (KRKL)



# Proteins analyzed: 1

# Number of proteins with cTP: 0 (0.0%)
# Number of proteins with cTP & possible mTP: 0 (0.0%)
# Number of proteins with cTP & NLS: 1 (100.0%)
# Number of proteins with cTP & possible mTP & NLS: 0 (0.0%)
# Number of proteins with mTP: 0 (0.0%)
# Number of proteins with mTP & possible cTP: 0 (0.0%)
# Number of proteins with mTP & NLS: 0 (0.0%)
# Number of proteins with mTP & possible cTP & NLS: 0 (0.0%)
# Number of proteins with NLS and no transit peptides: 0 (0.0%)

# Summary statistics

# Number of proteins with chloroplast localization (cTP, cTP & possible mTP, cTP & NLS, cTP & possible mTP & NLS): 1 (100.0%)
# Number of proteins with mitochondrial localization (mTP, mTP & possible cTP, mTP & NLS, mTP & possible cTP & NLS): 0 (0.0%)
# Number of proteins with nuclear localization and no transit peptides: 0 (0.0%)
# Number of proteins with nuclear localization and with transit peptides: 1 (100.0%)
```

Supplemental Figure 5. VviB6f is predicted to be secreted in plant chloroplasts. A, The protein sequence of VviB6f. Green represents chloroplast transit peptide. B, VviB6f has a chloroplast transit peptide. It shows that amino acids 1-41 is chloroplast transit peptide after the amino acid sequence of VviB6f is input into the software LOCALIZER.

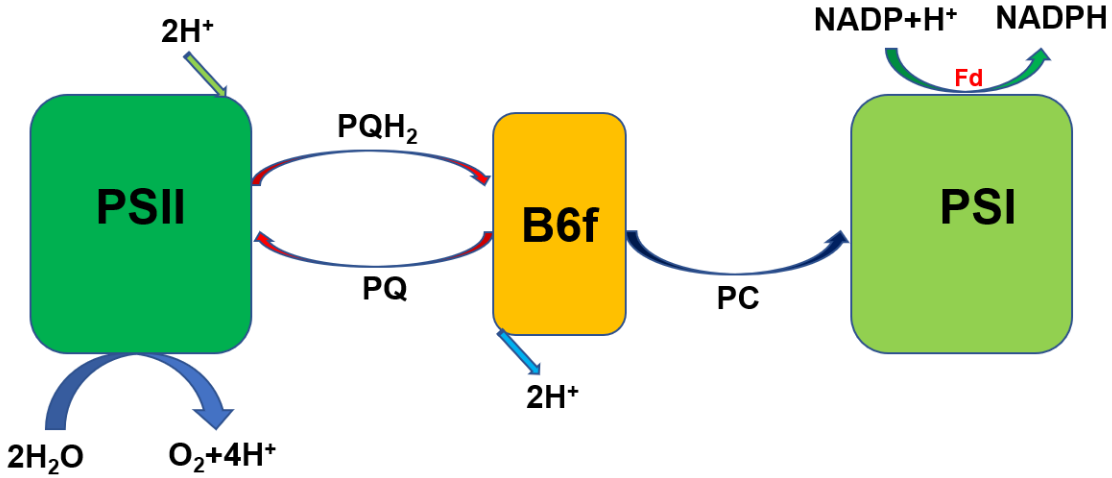

Supplemental Figure 6. B6f plays function in plant.

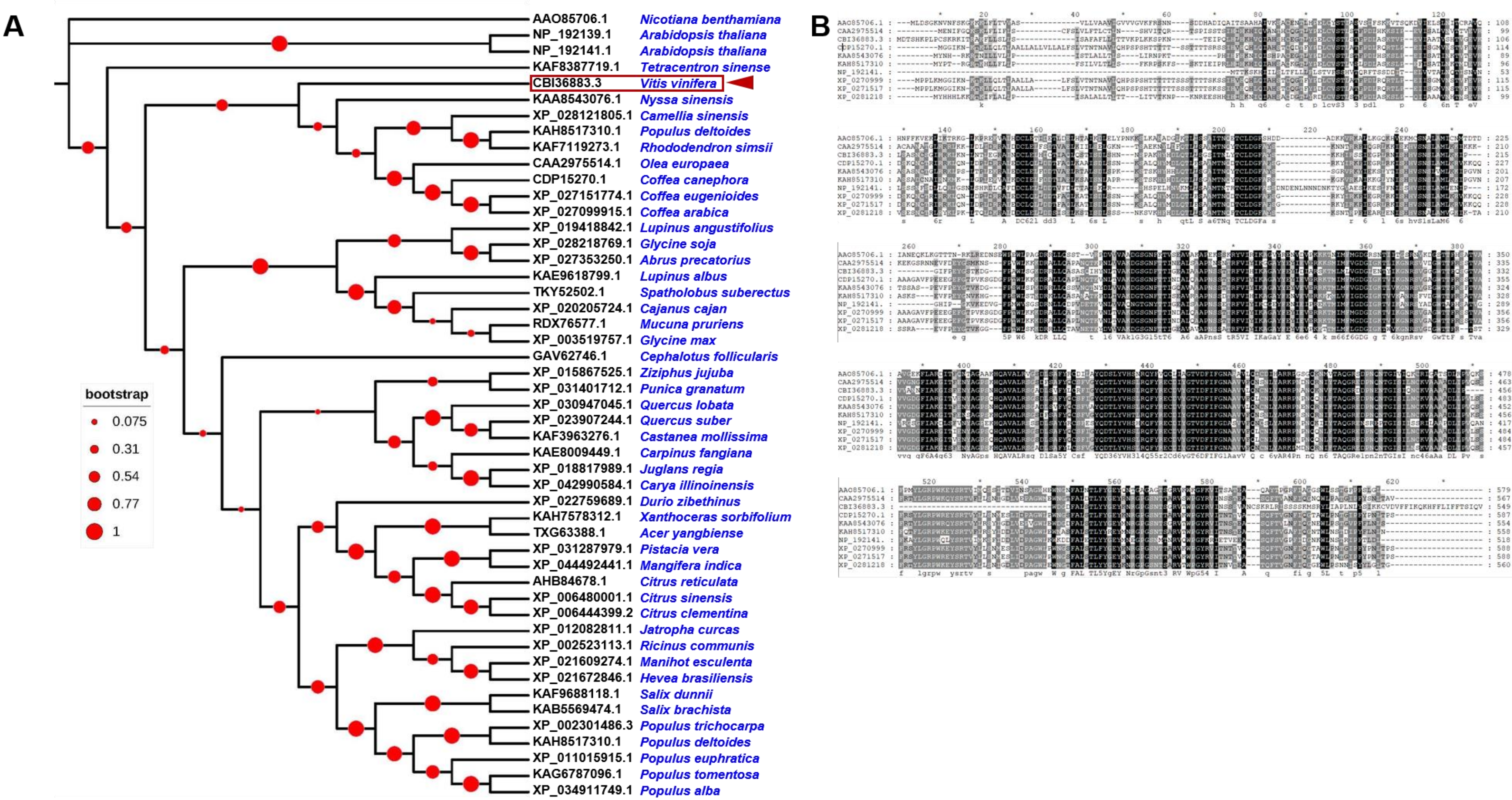

Supplemental Figure 7. The analysis of VviPE. A, Phylogenetic analysis of VviPE. Phylogenetic analysis with horticultural plants and model plant *Arabidopsis thaliana*, *Nicotiana benthamiana*, which was carried out by the MEGA6 software using the maximum likelihood method. The red triangle points to VviB6f. B, Sequence analysis of VviPE. The VviB6f amino acid sequences were alignment to plants that clustered in the same cluster downloaded from NCBI. B, Sequence analysis of VviPE. The VviPE amino acid sequences were alignment and downloed from NCBI.

Supplemental Table S1. CSEP080 screened proteins by yeast two-hybrid.

	Gene name	Gene ID
1	*Vitis vinifera* transcription factor ICE1	XM_034851681.1
2	*Vitis vinifera* eukaryotic translation initiation factor 3 subunit F	XM_034849562.1
3	*Vitis vinifera* acyl carrier protein 2	XM_003631834.3
4	*Vitis vinifera* VvPAL2	AB015871.1
5	*Vitis vinifera* beta-ureidopropionase-like	XM_034824904.1
6	*Vitis vinifera* protein yippee-like	XM_034821987.1
7	*Vitis vinifera* endoplasmic reticulum metallopeptidase 1	XM_034847951.1
8	*Vitis vinifera* pyrophosphate--fructose 6-phosphate 1-phosphotransferase	XM_002276233.3
9	*Vitis vinifera* bet1-like SNARE 1-1	XM_002285644.3
10	*Vitis vinifera* phospho-N-acetylmuramoyl-pentapeptide-transferase	XM_019222775.1
11	*Vitis vinifera* coatomer subunit beta-1-like	XM_002282374.3
12	*Vitis vinifera* DEAD-box ATP-dependent RNA helicase 10	XM_019223091.1
13	*Vitis vinifera* prosaposin-like	XM_034838487.1
14	*Vitis vinifera* E3 ubiquitin-protein ligase RGLG2	XM_034851097.1
15	*Vitis vinifera* protein arginine methyltransferase NDUFAF7	XM_019225361.1
16	*Vitis vinifera* polyubiquitin	XM_010646102.2
17	*Vitis vinifera* RNA-binding protein 1	XM_002275153.4
18	*Vitis vinifera* transcription factor EMB1444	XM_034834785.1
19	*Vitis vinifera* polyadenylate-binding protein RBP47B	XM_002285443.3
20	*Vitis vinifera* phosphomethylpyrimidine synthase	XM_004651534.1
21	*Vitis vinifera* mini-chromosome maintenance complex-binding protein	XM_034848413.1
22	*Vitis vinifera* thaumatin-like protein	XM_002282952.4
23	*Vitis vinifera* phosphatidylinositol glycan anchor biosynthesis class U protein-like	XM_034851901.1
24	*Vitis vinifera* protein LNK2	XM_002269785.4
25	*Vitis vinifera* acyl carrier protein 1	XM_034852500.1
26	*Vitis vinifera* rho guanine nucleotide exchange factor 8	XM_034843691.1
27	*Vitis vinifera* pectinesterase	XM_002271629.4
28	*Vitis vinifera* F-box protein PP2-B1-like	XM_034826868.1
29	*Vitis vinifera* 2-hydroxyisoflavanone dehydratase-like	XM_034830312.1
30	*Vitis vinifera* U-box domain-containing protein 32	XM_010664412.2
31	*Vitis vinifera* probable xyloglucan endotransglucosylase 7	XM_010648623.1
32	*Vitis vinifera* metal tolerance protein 11	XM_034849605.1
33	*Vitis vinifera* protein WVD2-like 5	XM_010650367.2
34	*Vitis vinifera* glutamyl-tRNA reductase 1	XM_002285547.3
35	*Vitis vinifera* NADP-dependent glyceraldehyde-3-phosphate dehydrogenase	XM_002285250.4
36	*Vitis vinifera* 3-hydroxyacyl-[acyl-carrier-protein] dehydratase FabZ-like	XM_034846048.1
37	*Vitis vinifera* tetraspanin-8-like	XM_034844164.1
38	*Vitis vinifera* pheophytinase	XM_002266319.4
39	*Vitis vinifera* mitochondrial outer membrane protein porin	XM_034841362.1
40	*Vitis vinifera* cytochrome b6-f complex iron-sulfur subunit	XM_034823548.1
41	*Vitis vinifera* glutamate decarboxylase	XM_034827506.1
42	*Vitis vinifera* gamma aminobutyrate transaminase 1	XM_002268767.4

Supplemental Table S2. Primers used in this study.

Primer Names	Primer Sequence
CSEP080-GFP-F	CACGGGGGACGAGCTCGGTACATGTGGCTCCAAACACGTGT
CSEP080-GFP-R	TCTAGAGGATCCCCGGGTCCACTCAGTTTTTTCGCATT
CSEP080-mCherry-F	CACGGGGGACGAGCTCGGTACATGTGGCTCCAAACACGTGT
CSEP080-mCherry-R	TCTAGAGGATCCCCGGGTCCACTCAGTTTTTTCGCATT
CSEP080SP-PSUC2-F	ATGAATTATGTGGCTCCAAACACGTGT
CSEP080SP-PSUC2-R	ATCTCGATCCCAAAACCATGGAGATAAAAAA
CSEP080ΔSP-PSUC2-F	ATGAATTGATCCAACCGAACAGACAATCAG
CSEP080ΔSP-PSUC2-R	ATCTCGACCACTCAGTTTTTTCGCATT
CSEP080-RNAi-F	TAGCATGGCCGCGGGATATCACAAGTTTGTACAAAAAAGCCCCTCGCTAAAACAGCTGCT
CSEP080-RNAi-R	GCGGCCGCACTAGTGATATCACCACTTTGTACAAGAAAGCCCACTCAGTTTTTTCGCATT
CSEP080-BD-F	ATGGCCATGGAGGCCGAATTCATGTGGCTCCAAACACGTGT
CSEP080-BD-R	GGCCGCTGCAGGTCGACGGATCCCCCACTCAGTTTTTTCGCATT
q-CSEP080-F	ATGTGGCTCCAAACACGTGT
q-CSEP080-R	CGTGAACACGCCGGAGCAGC
q-EF1-F	AAAGGATCATTCAAATATGC
q-EF1-R	GCAATGATTAAAATAGCACA
CSEP080-cYFP-F	GTACTGTCGACCTCGAGGGTACATGTGGCTCCAAACACGTGT
CSEP080-cYFP-R	TATGGGTACATCCCGGGAGCGGTCCACTCAGTTTTTTCGCATT
VviB6f-nYFP-F	ATCCGTCGACCTCGAGGGTACCATGGCTGCCTCCACTCTCTCCT
VviB6f-nYFP-R	TCGAGCTCCTACCCGGGAGCGGTCGGACCACCATGGATCTTCACCT
VviPE-nYFP-F	ATCCGTCGACCTCGAGGGTACCATGTACAACCACAGAGCAAGAG
VviPE-nYFP-R	TCGAGCTCCTACCCGGGAGCGGTCAACGATGGTCAAATTGGAATAA
CSEP080-nLuc-F	GAACACGGGGGACGAGCTCGGATGTGGCTCCAAACACGTGT
CSEP080-nLuc-R	GGGACGCGTACGAGATCTGGTCCCACTCAGTTTTTTCGCATT
VviB6f-cLuc-F	CTCGTACGCGTCCCGGGGCGGTATGGCTGCCTCCACTCTCTCCT
VviB6f-cLuc-R	GAACGAAAGCTCTGCAGGTCGAGGACCACCATGGATCTTCACCT
VviPE-cLuc-F	CTCGTACGCGTCCCGGGGCGGTATGTACAACCACAGAGCAAGAG
VviPE-cLuc-R	GAACGAAAGCTCTGCAGGTCGAAACGATGGTCAAATTGGAATAA
VviB6f-AD-F	GGTGGGCATCGATACGGGATCCATATGGCTGCCTCCACTCTCTC
VviB6f-AD-R	CTACGATTCATCTGCAGCTCGAGCGGACCACCATGGATCTTCAC
VviPE-AD-F	GGTGGGCATCGATACGGGATCCATATGTACAACCACAGAGCAAG
VviPE-AD-R	CTACGATTCATCTGCAGCTCGAGCAACGATGGTCAAATTGGAAT
VviB6f(N)-AD-F	GGTGGGCATCGATACGGGATCCATATGGCTGCCTCCACTCTCTC
VviB6f(N)-AD-R	CTACGATTCATCTGCAGCTCGAGCAATAACATCATTTCCAAGTG
VviB6f(C)-AD-F	GGTGGGCATCGATACGGGATCCATATGGCAGATGAATGGCTTAAGAC
VviB6f(C)-AD-R	CTACGATTCATCTGCAGCTCGAGCGGACCACCATGGATCTTCAC
VviPE(N)-AD-F	GGTGGGCATCGATACGGGATCCATATGTACAACCACAGAGCAAG
VviPE(N)-AD-R	CTACGATTCATCTGCAGCTCGAGCGTAATGAATTTGACTCGCCG
VviPE(C)-AD-F	GGTGGGCATCGATACGGGATCCATATGAATCTCACCGTGGCCAAGGA
VviPE(C)-AD-R	CTACGATTCATCTGCAGCTCGAGCAACGATGGTCAAATTGGAAT
VviB6f-GFP-F	CACGGGGGACGAGCTCGGTACATGGCTGCCTCCACTCTCTC
VviB6f-GFP-R	TCTAGAGGATCCCCGGGTGGACCACCATGGATCTTCAC
VviB6f-RNAi-F	TAGCATGGCCGCGGGATATCACAAGTTTGTACAAAAAAGCATGGCTGCCTCCACTCTCTCCT
VviB6f-RNAi-R	GCGGCCGCACTAGTGATATCACCACTTTGTACAAGAAAGCTCCTTGGCCACAATACCACC
VviPE-GFP-F	CACGGGGGACGAGCTCGGTACATGTACAACCACAGAGCAAG
VviPE-GFP-R	TCTAGAGGATCCCCGGGTAACGATGGTCAAATTGGAAT
VviPE-RNAi-F	TAGCATGGCCGCGGGATATCACAAGTTTGTACAAAAAAGCATGTACAACCACAGAGCAAG
VviPE-RNAi-R	GCGGCCGCACTAGTGATATCACCACTTTGTACAAGAAAGCCTCCGAATGCCGCTGCAGTT
VviB6f-luc-F	ACACGGGGGACGAGCTCGGTATGGCTGCCTCCACTCTCTC
VviB6f-luc-R	GCGTCTTCCATCCCGGGTACGGACCACCATGGATCTTCAC
VviPE-luc-F	ACACGGGGGACGAGCTCGGTATGTACAACCACAGAGCAAG
VviPE-luc-R	GCGTCTTCCATCCCGGGTACAACGATGGTCAAATTGGAAT
